# *N*,*N*′-[1,3-Phenyl­enebis(methyl­ene)]di-*p*-toluenesulfonamide

**DOI:** 10.1107/S1600536812007222

**Published:** 2012-03-03

**Authors:** Ejaz, Islam Ullah Khan, William T. A. Harrison, Rukhsana Anjum

**Affiliations:** a Materials Chemistry Laboratory, Department of Chemistry, GC University, Lahore 54000, Pakistan; b Department of Chemistry, University of Aberdeen, Meston Walk, Aberdeen AB24 3UE, Scotland; c Mediways International, 16 Km Multan Road, Lahore, Pakistan

## Abstract

In the title compound, C_22_H_24_N_2_O_4_S_2_, the dihedral angles between the central benzene ring and the pendant rings are 66.96 (13) and 69.37 (13)°. The torsion angles for the C—N—S—C fragments are −68.5 (3) and −72.6 (3)°. In the crystal, mol­ecules are linked by N—H⋯O hydrogen bonds to generate infinite (001) sheets containing *R*_4_^4^(28) loops. A weak aromatic π–π stacking contact between one of the terminal benzene rings and its inversion-related partner is also observed [centroid-to-centroid separation = 3.796 (2) Å and slippage = 1.581 Å], as are two possible C—H⋯π contacts.

## Related literature

For a related structure, see: Ejaz *et al.* (2011 ▶). For hydrogen-bonding motifs, see: Bernstein *et al.* (1995 ▶).
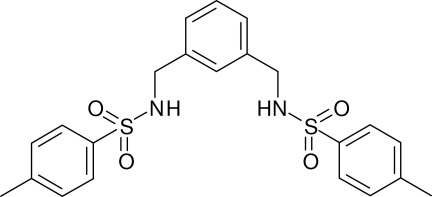


## Experimental

### 

#### Crystal data

C_22_H_24_N_2_O_4_S_2_
*M* *_r_* = 444.55
Triclinic, 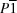

*a* = 7.7562 (3) Å
*b* = 8.7905 (3) Å
*c* = 17.1479 (5) Å
α = 87.554 (1)°
β = 86.151 (1)°
γ = 70.895 (1)°
*V* = 1102.00 (7) Å^3^
*Z* = 2
Mo *K*α radiation
μ = 0.27 mm^−1^
*T* = 296 K
0.30 × 0.20 × 0.15 mm

#### Data collection

Bruker APEXII CCD diffractometer
19493 measured reflections
5417 independent reflections
3937 reflections with *I* > 2σ(*I*)
*R*_int_ = 0.030

#### Refinement

*R*[*F* ^2^ > 2σ(*F* ^2^)] = 0.062
*wR*(*F* ^2^) = 0.187
*S* = 1.13
5417 reflections
273 parameters
H-atom parameters constrained
Δρ_max_ = 0.33 e Å^−3^
Δρ_min_ = −0.41 e Å^−3^

### 

Data collection: *APEX2* (Bruker, 2007 ▶); cell refinement: *SAINT* (Bruker, 2007 ▶); data reduction: *SAINT*; program(s) used to solve structure: *SHELXS97* (Sheldrick, 2008 ▶); program(s) used to refine structure: *SHELXL97* (Sheldrick, 2008 ▶); molecular graphics: *ORTEP-3* (Farrugia, 1997 ▶); software used to prepare material for publication: *SHELXL97*.

## Supplementary Material

Crystal structure: contains datablock(s) I, global. DOI: 10.1107/S1600536812007222/su2379sup1.cif

Structure factors: contains datablock(s) I. DOI: 10.1107/S1600536812007222/su2379Isup2.hkl

Supplementary material file. DOI: 10.1107/S1600536812007222/su2379Isup3.cml

Additional supplementary materials:  crystallographic information; 3D view; checkCIF report

## Figures and Tables

**Table 1:** Hydrogen-bond geometry (Å, °) *Cg*1 is the centroid of the C1–C6 ring and *Cg*2 is the centroid of the C9–C14 ring.

*D*—H⋯*A*	*D*—H	H⋯*A*	*D*⋯*A*	*D*—H⋯*A*
N1—H1⋯O4^i^	0.93	2.02	2.896 (4)	156
N2—H2⋯O2^ii^	0.93	2.09	2.989 (4)	165
C7—H7*A*⋯*Cg*1^iii^	0.96	2.74	3.560 (5)	144
C21—H21⋯*Cg*2^iv^	0.93	2.74	3.560 (4)	147

Symmetry codes: (i) 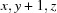; (ii) 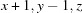; (iii) 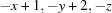; (iv) 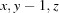.

**Fig. 1.**
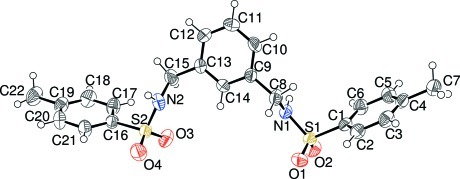
The molecular structure of the title molecule, showing the atom numbering and the displacement ellipsoids drawn at the 50% probability level.

**Fig. 2.**
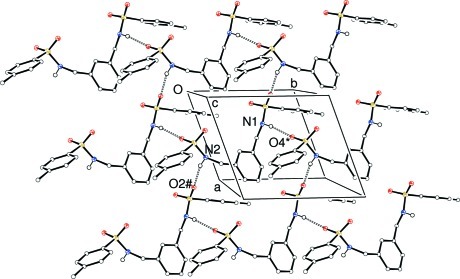
Fragment of a (001) sheet of molecules of the title compound, linked by N—H···O hydrogen bonds [double dashed lines; C-bound H atoms have been omitted for clarity; O4* and O2# are at symmetry positions (x, y + 1, z) and (x + 1, y - 1, z), respectively].

## supplementary crystallographic information

### Comment

As part of our ongoing structural studies of symmetric aromatic sulfonamides (Ejaz *et al.*, 2011), the synthesis and crystal structure of the title compound are described herein.

In the molecule of the title compound (Fig. 1), the dihedral angle between the central (C9) benzene ring and the C1- and C16-pendant rings are 66.96 (13) and 69.37 (13)°, respectively. The C1- and C16- pendant rings are inclined to one another by 31.8 (2)°. The conformations of the C1—S1—N1—C8 and C16—S2—N2—C15 fragments are both approximately *gauche* [torsion angles = -68.5 (3) and -72.6 (3)°, respectively], and the molecule has approximate non-crystallographic twofold symmetry about the axis passing through atoms C11 and C14.

In the crystal, the molecules are linked by N—H···O hydrogen bonds (Table 1), to generate (001) sheets (Fig. 2). The smallest identifiable circuit in the sheets is an *R*_4_^4^(28) loop. A weak aromatic π–π stacking contact between the C1-benzene ring and its inversion related partner at (-*x*, 2 - *y*, -*z*) is also observed [centroid-centroid separation = 3.796 (2) Å, slippage = 1.581 Å], as are two possible C—H···π contacts (Table 1).

The crystal structure of the diphenyl analogue has been reported on recently (Ejaz *et al.*, 2011). There the complete molecule is generated by crystallographic twofold symmetry, but its intermolecular linkages (a layered network constructed from N—H···O hydrogen bonds supplemented by C—H···π and π–π contacts) are very similar to those seen in the crystal structure of the title compound.

### Experimental

Benzene-1,3-diyldimethanamine (0.132 ml, 1.0 mmol) was mixed with 25 ml distilled water in a 50 ml round-bottom flask. 4-Methyl benzene sulfonyl chloride (0.38 g, 2.0 mmol) was added while maintaining the pH of the reaction mixture at 9.0 using 3% sodium carbonate solution. The suspension was stirred for about four hours and a white product was filtered, washed, dried and recrystallized from methanol to yield colourless crystalline flakes and chips of the title compound.

### Refinement

The H atoms were placed in calculated positions (N—H = 0.93 Å; C—H = 0.93–0.97 Å) and refined as riding atoms with *U*_iso_(H) = 1.2*U*_eq_(C,N) or 1.5*U*_eq_(methyl C).

### Crystal data

**Table:**

C_22_H_24_N_2_O_4_S_2_	*Z* = 2
*M**_r_* = 444.55	*F*(000) = 468
Triclinic, *P*1	*D*_x_ = 1.340 Mg m^−^^3^
Hall symbol: -P 1	Mo *K*α radiation, λ = 0.71073 Å
*a* = 7.7562 (3) Å	Cell parameters from 6272 reflections
*b* = 8.7905 (3) Å	θ = 2.7–27.1°
*c* = 17.1479 (5) Å	µ = 0.27 mm^−^^1^
α = 87.554 (1)°	*T* = 296 K
β = 86.151 (1)°	Chip, colourless
γ = 70.895 (1)°	0.30 × 0.20 × 0.15 mm
*V* = 1102.00 (7) Å^3^	

### Data collection

**Table:**

Bruker APEXII CCD diffractometer	3937 reflections with *I* > 2σ(*I*)
Radiation source: fine-focus sealed tube	*R*_int_ = 0.030
Graphite monochromator	θ_max_ = 28.4°, θ_min_ = 2.7°
ω scans	*h* = −10→6
19493 measured reflections	*k* = −11→9
5417 independent reflections	*l* = −22→21

### Refinement

**Table:**

Refinement on *F*^2^	Primary atom site location: structure-invariant direct methods
Least-squares matrix: full	Secondary atom site location: difference Fourier map
*R*[*F*^2^ > 2σ(*F*^2^)] = 0.062	Hydrogen site location: inferred from neighbouring sites
*wR*(*F*^2^) = 0.187	H-atom parameters constrained
*S* = 1.13	*w* = 1/[σ^2^(*F*_o_^2^) + (0.0628*P*)^2^ + 1.4755*P*] where *P* = (*F*_o_^2^ + 2*F*_c_^2^)/3
5417 reflections	(Δ/σ)_max_ = 0.002
273 parameters	Δρ_max_ = 0.33 e Å^−^^3^
0 restraints	Δρ_min_ = −0.41 e Å^−^^3^

### Special details

**Table:**

Geometry. All e.s.d.'s (except the e.s.d. in the dihedral angle between two l.s. planes) are estimated using the full covariance matrix. The cell e.s.d.'s are taken into account individually in the estimation of e.s.d.'s in distances, angles and torsion angles; correlations between e.s.d.'s in cell parameters are only used when they are defined by crystal symmetry. An approximate (isotropic) treatment of cell e.s.d.'s is used for estimating e.s.d.'s involving l.s. planes.
Refinement. Refinement of *F*^2^ against ALL reflections. The weighted *R*-factor *wR* and goodness of fit *S* are based on *F*^2^, conventional *R*-factors *R* are based on *F*, with *F* set to zero for negative *F*^2^. The threshold expression of *F*^2^ > σ(*F*^2^) is used only for calculating *R*-factors(gt) *etc*. and is not relevant to the choice of reflections for refinement. *R*-factors based on *F*^2^ are statistically about twice as large as those based on *F*, and *R*-factors based on ALL data will be even larger.

### Fractional atomic coordinates and isotropic or equivalent isotropic displacement parameters (Å^2^)

**Table:**

	*x*	*y*	*z*	*U*_iso_*/*U*_eq_	
C1	0.2038 (4)	0.8090 (4)	0.09032 (18)	0.0368 (7)	
C2	0.2158 (5)	0.8022 (4)	0.0097 (2)	0.0428 (7)	
H2A	0.2057	0.7136	−0.0150	0.051*	
C3	0.2430 (5)	0.9293 (4)	−0.0337 (2)	0.0480 (8)	
H3	0.2527	0.9247	−0.0880	0.058*	
C4	0.2561 (4)	1.0631 (4)	0.0017 (2)	0.0458 (8)	
C5	0.2419 (5)	1.0681 (4)	0.0823 (2)	0.0522 (9)	
H5	0.2502	1.1573	0.1070	0.063*	
C6	0.2152 (5)	0.9410 (4)	0.1267 (2)	0.0467 (8)	
H6	0.2050	0.9454	0.1809	0.056*	
C7	0.2849 (6)	1.2011 (5)	−0.0458 (3)	0.0642 (11)	
H7A	0.4002	1.2116	−0.0348	0.096*	
H7B	0.1884	1.2987	−0.0326	0.096*	
H7C	0.2846	1.1814	−0.1004	0.096*	
C8	0.5167 (5)	0.4630 (4)	0.1421 (2)	0.0448 (8)	
H8A	0.5583	0.5366	0.1081	0.054*	
H8B	0.4842	0.3902	0.1095	0.054*	
C9	0.6684 (4)	0.3680 (4)	0.19293 (19)	0.0376 (7)	
C10	0.8289 (5)	0.4027 (4)	0.1920 (2)	0.0486 (9)	
H10	0.8447	0.4853	0.1597	0.058*	
C11	0.9671 (5)	0.3151 (5)	0.2390 (2)	0.0545 (9)	
H11	1.0748	0.3402	0.2386	0.065*	
C12	0.9465 (5)	0.1917 (4)	0.2862 (2)	0.0471 (8)	
H12	1.0406	0.1322	0.3171	0.057*	
C13	0.7851 (5)	0.1558 (4)	0.2878 (2)	0.0407 (7)	
C14	0.6483 (4)	0.2438 (4)	0.24124 (19)	0.0395 (7)	
H14	0.5401	0.2195	0.2421	0.047*	
C15	0.7604 (6)	0.0222 (5)	0.3412 (2)	0.0546 (10)	
H15A	0.8756	−0.0365	0.3637	0.066*	
H15B	0.6719	0.0684	0.3836	0.066*	
C16	0.6242 (4)	−0.2929 (4)	0.41314 (19)	0.0391 (7)	
C17	0.6438 (5)	−0.2385 (4)	0.4858 (2)	0.0509 (9)	
H17	0.6070	−0.1286	0.4947	0.061*	
C18	0.7171 (6)	−0.3463 (5)	0.5445 (2)	0.0563 (10)	
H18	0.7304	−0.3086	0.5928	0.068*	
C19	0.7714 (5)	−0.5093 (5)	0.5330 (2)	0.0514 (9)	
C20	0.7504 (6)	−0.5623 (4)	0.4604 (2)	0.0569 (10)	
H20	0.7865	−0.6723	0.4518	0.068*	
C21	0.6772 (6)	−0.4554 (4)	0.4005 (2)	0.0506 (9)	
H21	0.6640	−0.4930	0.3522	0.061*	
C22	0.8531 (6)	−0.6281 (6)	0.5971 (3)	0.0731 (13)	
H22A	0.9644	−0.6136	0.6113	0.110*	
H22B	0.8789	−0.7357	0.5791	0.110*	
H22C	0.7683	−0.6109	0.6419	0.110*	
S1	0.16991 (10)	0.64814 (10)	0.14733 (5)	0.0395 (2)	
S2	0.53793 (12)	−0.15372 (10)	0.33690 (5)	0.0422 (2)	
N1	0.3566 (4)	0.5539 (3)	0.19015 (16)	0.0391 (6)	
H1	0.3810	0.6124	0.2295	0.047*	
N2	0.6978 (4)	−0.0890 (3)	0.29893 (18)	0.0478 (7)	
H2	0.7907	−0.1623	0.2698	0.057*	
O1	0.1399 (4)	0.5365 (3)	0.09599 (16)	0.0537 (6)	
O2	0.0363 (3)	0.7163 (3)	0.20915 (15)	0.0567 (7)	
O3	0.3995 (4)	−0.0193 (3)	0.37070 (17)	0.0614 (7)	
O4	0.4957 (4)	−0.2391 (3)	0.27635 (16)	0.0632 (8)	

### Atomic displacement parameters (Å^2^)

**Table:**

	*U*^11^	*U*^22^	*U*^33^	*U*^12^	*U*^13^	*U*^23^
C1	0.0298 (14)	0.0401 (17)	0.0370 (17)	−0.0068 (12)	−0.0034 (12)	0.0024 (13)
C2	0.0409 (17)	0.0444 (18)	0.0405 (18)	−0.0102 (14)	−0.0028 (14)	−0.0021 (15)
C3	0.0451 (19)	0.057 (2)	0.0354 (18)	−0.0097 (16)	−0.0012 (14)	0.0059 (16)
C4	0.0320 (16)	0.0459 (19)	0.054 (2)	−0.0058 (14)	−0.0060 (14)	0.0135 (16)
C5	0.058 (2)	0.0424 (19)	0.057 (2)	−0.0172 (17)	−0.0097 (18)	0.0017 (17)
C6	0.055 (2)	0.0461 (19)	0.0384 (18)	−0.0157 (16)	−0.0055 (15)	0.0016 (15)
C7	0.050 (2)	0.063 (3)	0.078 (3)	−0.0178 (19)	−0.012 (2)	0.028 (2)
C8	0.0429 (18)	0.0429 (18)	0.0425 (19)	−0.0073 (15)	−0.0009 (14)	0.0094 (15)
C9	0.0357 (15)	0.0313 (15)	0.0422 (17)	−0.0069 (12)	0.0005 (13)	0.0028 (13)
C10	0.0463 (19)	0.0418 (18)	0.060 (2)	−0.0200 (15)	0.0047 (16)	0.0113 (16)
C11	0.0389 (18)	0.058 (2)	0.072 (3)	−0.0240 (17)	−0.0011 (17)	0.0060 (19)
C12	0.0354 (16)	0.0439 (19)	0.059 (2)	−0.0074 (14)	−0.0092 (15)	0.0027 (16)
C13	0.0445 (17)	0.0323 (16)	0.0450 (18)	−0.0124 (13)	−0.0045 (14)	0.0063 (14)
C14	0.0376 (16)	0.0388 (17)	0.0450 (18)	−0.0168 (13)	−0.0029 (13)	0.0042 (14)
C15	0.071 (3)	0.049 (2)	0.051 (2)	−0.0285 (19)	−0.0196 (19)	0.0165 (17)
C16	0.0377 (16)	0.0363 (16)	0.0417 (18)	−0.0101 (13)	−0.0017 (13)	0.0024 (14)
C17	0.061 (2)	0.0413 (19)	0.043 (2)	−0.0053 (16)	−0.0046 (16)	−0.0032 (15)
C18	0.066 (2)	0.063 (2)	0.0352 (19)	−0.015 (2)	−0.0035 (17)	−0.0012 (17)
C19	0.0461 (19)	0.055 (2)	0.047 (2)	−0.0115 (17)	0.0017 (16)	0.0169 (17)
C20	0.070 (3)	0.0349 (18)	0.060 (2)	−0.0101 (17)	−0.005 (2)	0.0076 (17)
C21	0.067 (2)	0.0373 (18)	0.047 (2)	−0.0151 (17)	−0.0100 (17)	0.0023 (15)
C22	0.067 (3)	0.080 (3)	0.062 (3)	−0.015 (2)	−0.008 (2)	0.032 (2)
S1	0.0328 (4)	0.0434 (5)	0.0423 (5)	−0.0134 (3)	−0.0019 (3)	0.0049 (3)
S2	0.0466 (5)	0.0346 (4)	0.0440 (5)	−0.0101 (3)	−0.0110 (4)	0.0035 (3)
N1	0.0382 (14)	0.0368 (14)	0.0392 (15)	−0.0082 (11)	−0.0049 (11)	0.0043 (11)
N2	0.0591 (18)	0.0344 (14)	0.0495 (17)	−0.0160 (13)	0.0012 (14)	0.0055 (13)
O1	0.0555 (15)	0.0546 (15)	0.0606 (16)	−0.0288 (12)	−0.0140 (12)	0.0003 (12)
O2	0.0398 (13)	0.0673 (17)	0.0541 (15)	−0.0089 (12)	0.0101 (11)	0.0076 (13)
O3	0.0505 (15)	0.0505 (15)	0.0697 (18)	0.0019 (12)	−0.0049 (13)	0.0029 (13)
O4	0.089 (2)	0.0570 (16)	0.0530 (16)	−0.0332 (15)	−0.0285 (15)	0.0058 (13)

### Geometric parameters (Å, º)

**Table:**

C1—C6	1.371 (5)	C13—C15	1.512 (5)
C1—C2	1.382 (5)	C14—H14	0.9300
C1—S1	1.765 (3)	C15—N2	1.461 (5)
C2—C3	1.382 (5)	C15—H15A	0.9700
C2—H2A	0.9300	C15—H15B	0.9700
C3—C4	1.381 (5)	C16—C21	1.374 (5)
C3—H3	0.9300	C16—C17	1.388 (5)
C4—C5	1.381 (5)	C16—S2	1.760 (3)
C4—C7	1.502 (5)	C17—C18	1.370 (5)
C5—C6	1.389 (5)	C17—H17	0.9300
C5—H5	0.9300	C18—C19	1.374 (6)
C6—H6	0.9300	C18—H18	0.9300
C7—H7A	0.9600	C19—C20	1.385 (6)
C7—H7B	0.9600	C19—C22	1.505 (5)
C7—H7C	0.9600	C20—C21	1.381 (5)
C8—N1	1.459 (4)	C20—H20	0.9300
C8—C9	1.508 (4)	C21—H21	0.9300
C8—H8A	0.9700	C22—H22A	0.9600
C8—H8B	0.9700	C22—H22B	0.9600
C9—C10	1.374 (5)	C22—H22C	0.9600
C9—C14	1.386 (4)	S1—O1	1.429 (3)
C10—C11	1.384 (5)	S1—O2	1.434 (3)
C10—H10	0.9300	S1—N1	1.621 (3)
C11—C12	1.371 (5)	S2—O4	1.422 (3)
C11—H11	0.9300	S2—O3	1.424 (3)
C12—C13	1.386 (5)	S2—N2	1.614 (3)
C12—H12	0.9300	N1—H1	0.9305
C13—C14	1.373 (4)	N2—H2	0.9265
			
C6—C1—C2	120.5 (3)	N2—C15—C13	111.3 (3)
C6—C1—S1	119.5 (3)	N2—C15—H15A	109.4
C2—C1—S1	120.0 (3)	C13—C15—H15A	109.4
C3—C2—C1	118.9 (3)	N2—C15—H15B	109.4
C3—C2—H2A	120.5	C13—C15—H15B	109.4
C1—C2—H2A	120.5	H15A—C15—H15B	108.0
C4—C3—C2	121.5 (3)	C21—C16—C17	119.7 (3)
C4—C3—H3	119.2	C21—C16—S2	120.3 (3)
C2—C3—H3	119.2	C17—C16—S2	120.0 (3)
C3—C4—C5	118.6 (3)	C18—C17—C16	120.2 (3)
C3—C4—C7	121.3 (4)	C18—C17—H17	119.9
C5—C4—C7	120.1 (4)	C16—C17—H17	119.9
C4—C5—C6	120.5 (3)	C17—C18—C19	121.0 (4)
C4—C5—H5	119.7	C17—C18—H18	119.5
C6—C5—H5	119.7	C19—C18—H18	119.5
C1—C6—C5	119.8 (3)	C18—C19—C20	118.3 (3)
C1—C6—H6	120.1	C18—C19—C22	121.2 (4)
C5—C6—H6	120.1	C20—C19—C22	120.5 (4)
C4—C7—H7A	109.5	C21—C20—C19	121.5 (3)
C4—C7—H7B	109.5	C21—C20—H20	119.3
H7A—C7—H7B	109.5	C19—C20—H20	119.3
C4—C7—H7C	109.5	C16—C21—C20	119.3 (4)
H7A—C7—H7C	109.5	C16—C21—H21	120.4
H7B—C7—H7C	109.5	C20—C21—H21	120.4
N1—C8—C9	110.5 (3)	C19—C22—H22A	109.5
N1—C8—H8A	109.6	C19—C22—H22B	109.5
C9—C8—H8A	109.6	H22A—C22—H22B	109.5
N1—C8—H8B	109.6	C19—C22—H22C	109.5
C9—C8—H8B	109.6	H22A—C22—H22C	109.5
H8A—C8—H8B	108.1	H22B—C22—H22C	109.5
C10—C9—C14	118.9 (3)	O1—S1—O2	119.71 (17)
C10—C9—C8	120.7 (3)	O1—S1—N1	106.78 (15)
C14—C9—C8	120.4 (3)	O2—S1—N1	105.48 (15)
C9—C10—C11	120.3 (3)	O1—S1—C1	108.30 (16)
C9—C10—H10	119.9	O2—S1—C1	107.56 (15)
C11—C10—H10	119.9	N1—S1—C1	108.60 (14)
C12—C11—C10	120.4 (3)	O4—S2—O3	119.86 (18)
C12—C11—H11	119.8	O4—S2—N2	105.13 (17)
C10—C11—H11	119.8	O3—S2—N2	107.23 (17)
C11—C12—C13	119.9 (3)	O4—S2—C16	107.55 (16)
C11—C12—H12	120.1	O3—S2—C16	107.40 (17)
C13—C12—H12	120.1	N2—S2—C16	109.40 (16)
C14—C13—C12	119.3 (3)	C8—N1—S1	118.4 (2)
C14—C13—C15	120.7 (3)	C8—N1—H1	114.7
C12—C13—C15	120.0 (3)	S1—N1—H1	113.5
C13—C14—C9	121.2 (3)	C15—N2—S2	120.7 (3)
C13—C14—H14	119.4	C15—N2—H2	113.3
C9—C14—H14	119.4	S2—N2—H2	116.1
			
C6—C1—C2—C3	1.3 (5)	C17—C18—C19—C20	0.1 (6)
S1—C1—C2—C3	−179.8 (3)	C17—C18—C19—C22	179.6 (4)
C1—C2—C3—C4	−0.8 (5)	C18—C19—C20—C21	0.0 (6)
C2—C3—C4—C5	0.2 (5)	C22—C19—C20—C21	−179.4 (4)
C2—C3—C4—C7	−179.8 (3)	C17—C16—C21—C20	−0.4 (6)
C3—C4—C5—C6	0.1 (5)	S2—C16—C21—C20	177.8 (3)
C7—C4—C5—C6	−180.0 (3)	C19—C20—C21—C16	0.1 (6)
C2—C1—C6—C5	−1.0 (5)	C6—C1—S1—O1	172.7 (3)
S1—C1—C6—C5	−180.0 (3)	C2—C1—S1—O1	−6.2 (3)
C4—C5—C6—C1	0.4 (6)	C6—C1—S1—O2	42.0 (3)
N1—C8—C9—C10	−114.7 (4)	C2—C1—S1—O2	−136.9 (3)
N1—C8—C9—C14	65.5 (4)	C6—C1—S1—N1	−71.7 (3)
C14—C9—C10—C11	−0.4 (5)	C2—C1—S1—N1	109.4 (3)
C8—C9—C10—C11	179.8 (3)	C21—C16—S2—O4	14.3 (4)
C9—C10—C11—C12	0.9 (6)	C17—C16—S2—O4	−167.6 (3)
C10—C11—C12—C13	−1.0 (6)	C21—C16—S2—O3	144.5 (3)
C11—C12—C13—C14	0.7 (6)	C17—C16—S2—O3	−37.3 (3)
C11—C12—C13—C15	−178.6 (4)	C21—C16—S2—N2	−99.4 (3)
C12—C13—C14—C9	−0.2 (5)	C17—C16—S2—N2	78.8 (3)
C15—C13—C14—C9	179.1 (3)	C9—C8—N1—S1	−173.1 (2)
C10—C9—C14—C13	0.1 (5)	O1—S1—N1—C8	48.1 (3)
C8—C9—C14—C13	179.9 (3)	O2—S1—N1—C8	176.5 (2)
C14—C13—C15—N2	50.8 (5)	C1—S1—N1—C8	−68.5 (3)
C12—C13—C15—N2	−129.9 (4)	C13—C15—N2—S2	−136.6 (3)
C21—C16—C17—C18	0.6 (6)	O4—S2—N2—C15	172.2 (3)
S2—C16—C17—C18	−177.6 (3)	O3—S2—N2—C15	43.6 (3)
C16—C17—C18—C19	−0.4 (6)	C16—S2—N2—C15	−72.6 (3)

### Hydrogen-bond geometry (Å, º)

Cg1 is the centroid of the C1–C6 ring and Cg2 is the centroid of the C9–C14 ring.

**Table:**

*D*—H···*A*	*D*—H	H···*A*	*D*···*A*	*D*—H···*A*
N1—H1···O4^i^	0.93	2.02	2.896 (4)	156
N2—H2···O2^ii^	0.93	2.09	2.989 (4)	165
C7—H7*A*···*Cg*1^iii^	0.96	2.74	3.560 (5)	144
C21—H21···*Cg*2^iv^	0.93	2.74	3.560 (4)	147

Symmetry codes: (i) *x*, *y*+1, *z*; (ii) *x*+1, *y*−1, *z*; (iii) −*x*+1, −*y*+2, −*z*; (iv) *x*, *y*−1, *z*.
